# Monoclonal Antibodies Against the Calcitonin Gene-Related Peptide and Its Receptor in Japanese Adolescents With Migraines

**DOI:** 10.7759/cureus.33689

**Published:** 2023-01-12

**Authors:** Masahito Katsuki, Kenta Kashiwagi, Shin Kawamura, Akihito Koh

**Affiliations:** 1 Department of Neurosurgery, Itoigawa General Hospital, Itoigawa, JPN; 2 Department of Neurology, Itoigawa General Hospital, Itoigawa, JPN

**Keywords:** preventive therapy, headache, adolescents, migraine, calcitonin gene-related peptide-related monoclonal antibodies

## Abstract

Introduction

Adolescent migraines is a public health problem, and effective prophylactic treatment is needed. In Japan, three types of calcitonin gene-related peptide-related monoclonal antibodies (CGRP-mABs) are available. Galcanezumab, fremanezumab, and erenumab can be used for migraine prevention in ages 15 years or older, but reports on adolescent migraine treated with CGRP-mABs remain few. We described this study to report the real-world data of CGRP-mABs' efficacy for adolescents with migraines aged from 15 to 17 years old.

Methods

We retrospectively investigated ten adolescent migraine patients aged from 15 to 17 years old treated with CGRP-mABs. Headache impact test-6 (HIT-6), monthly headache days (MHD), and monthly acute medication intake days (AMD) before and three months after CGRP-mABs treatment were evaluated.

Results

Six females and four males were included. Seven had episodic migraines (EM), three had EM and tension-type headaches, one had chronic migraines (CM), and one had CM and medication-overuse headaches. As chief obstacles to life due to headaches, five reported them as detrimental to study, one reported them as detrimental to playing sports, and four reported missing school. The median HIT-6 was 63 (46-68) and 44 (36-65) before and three months after treatment, respectively. Median of MHD was 5.5 (1-29) and 1.5 (1-30), respectively, and the median of AMD was 5.5 (1-30) and 1 (0-30), respectively. A significant reduction of HIT-6 was observed at three months (p=0.008). Six (60%) of the ten patients experienced therapeutic effectiveness. Patients with missing school as the chief obstacle to life due to headaches seemed ineffective compared to those with other obstacles (p=0.048). There were no side effects of CGRP-mABs.

Conclusion

We herein described the ten adolescent migraine patients treated with CGRP-mABs. HIT-6 score significantly decreased at three months, and six of the ten patients experienced therapeutic effectiveness measured by HIT-6. Now several trials have been ongoing to test the efficacy of CGRP-mABs for adolescents. Urgent evidence accumulation is needed about CGRP-mABs for adolescents.

## Introduction

Migraines are a widespread public health problem [1]. In Japan, migraine prevalence is 4.3-8.4% [2-4], and 29.8-74.2% of patients with migraine headaches report that it significantly impairs their daily activity [1]. Also, the migraine prevalence among adolescents aged 15 to 17 years old is 16.2% and those of medication-overuse headache (MOH) is 1.5% in Japan [5]. Effective prophylactic treatment for adolescents with migraine is needed. However, the classical prophylactic treatment has limited efficacy and is sometimes associated with side effects that can result in poor adherence [6,7]. Most of the randomized controlled studies on migraine-prophylactic drugs prescribed for adults could not show superiority to placebos in adolescents with migraines [8]. In this context, effective prophylactic medication for migraine with good adherence is needed.

In Japan, three types of calcitonin gene-related peptide (CGRP)-related monoclonal antibodies (CGRP-mABs), galcanezumab, fremanezumab, and erenumab, can be used for migraine prevention in adult patients aged 18 years or older. There has been a growing interest in their application in adolescent migraine treatment. The American Headache Society released recommendations on the use of CGRP-mABs in children and adolescents with a full review of existing prophylactic drugs and caution about CGRP-mABs [9]. According to the Japanese Pharmaceutical Affairs Law, an adult is 15 years of age or older, and CGRP-mABs can be used if the patient is 15 years of age or older. We herein presented 10 adolescent migraine patients aged from 15 to 17 years old treated with CGRP-mABs, as an early clinical experience.

## Materials and methods

Patient population

We retrospectively investigated medical records between April 2021 and December 2022. Ten adolescent migraine patients aged from 15 to 17 years old who were treated with CGRP-mABs at our hospital were included. The patients had headaches at least 90 days before the CGRP-mAbs treatment, and had been keeping headache diaries. They had had at least one type of prophylactic medication before CGRP-mABs use, but it failed. We made the headache diagnosis according to the International Classifications of Headache Disorder third edition [10]. Chronic migraine (CM), episodic migraine (EM), tension-type headache (TTH), and MOH were diagnosed. Written informed consent was obtained for this study from all the patients or patients' families.

Investigated variables and outcomes

We collected patients' characteristics, such as age, sex, medical history, chief obstacles to life due to headaches, and previous prophylactic medication. Clinical data reported by paper-based or electronic headache diaries were used. Monthly headache days (MHD) and monthly acute medication intake days (AMD) were defined as the monthly values over the respective observation period of 30 days. Head Impact Test-6 (HIT-6) [11] was also investigated over the respective observation period. The outcomes were defined as the changes in HIT-6, MHD, and AMD before treatment and after three months. We defined more than 50% reduction in MHD at three months, or a three-month HIT-6 score under 50, as therapeutically effective.

Statistical analysis

Results were presented as median (range). Wilcoxon's test was performed to compare HIT-6, MHD, and AMD before treatment and after three months. Fisher's exact test tested the relationship between therapeutically effective and the chief obstacles to life. We conducted these analyses using version 28.0.0 of SPSS software (IBM Inc., Armonk, New York). A two-tailed p-value of <0.05 was considered statistically significant.

## Results

Table 1 shows the characteristics of the ten adolescent migraine patients. Six females and four males were included. Of the ten patients, seven had EM, three had EM+TTH, one had CM, and one had CM+MOH. As chief obstacles to life due to headaches, five reported them as detrimental to study, one reported them as detrimental to playing sports, and four reported missing school. Patient five initially took 675 mg fremanezumab, but it was not effective. Erenumab was then started, but it failed. Patient eight initially took fremanezumab, and it was effective. Since she had difficulty taking time off from school to make regular visits to the hospital to take 675 mg fremanezumab, she started galcanezumab, which can be self-injected. Other details are described in Table 1.

**Table 1:** Patient characteristics AMD - monthly acute medication intake days, CGRP-mABs - calcitonin gene-related peptide-related monoclonal antibodies, CM - chronic migraine, EM - episodic migraine, HIT-6 - Headache Impact Test-6, MHD - monthly headache days, MOH - medication-overuse headache, TTH - tension-type headache.

No.	Age	Sex	Diagnosis	Main obstacles to life	Medical history	Previous prophylactic medication	CGRP-mABs	MHD	AMD	HIT-6	3-month MHD	3-month AMD	3-month HIT-6	Continuation period
1	15	Female	EM+TTH	Detrimental to study	Epilepsy	Topiramate	Galcanezumab	11	11	56	2	2	42	8m
2	15	Male	EM	Detrimental to study	-	Kampo (goreisan)	Galcanezumab	6	6	68	1	1	48	3m
3	15	Female	CM+MOH	Missing school	Hyperthyroidism	Amitriptyline	Galcanezumab	29	29	65	0	0	36	6m
4	15	Female	CM	Missing school	-	Kampo (goreisan)	Galcanezumab	28	14	67	21	8	60	3m
5-1	16	Male	EM	Missing school	-	Valproic acid	Fremanezumab 675mg	4	4	65	12	12	62	3m, switched to erenumab.
5-2	16	Male	EM+TTH	Missing school	-	Valproic acid, fremanezumab	Switch to erenumab	12	12	62	0	0	40	3m, stopped. Changed school and treated at psychiatry.
6	16	Female	EM	Missing school	Dysmenorrhea	Amitriptyline, low-dose pill	Erenumab	4	4	67	6	6	65	3m, stopped. Treated as orthostatic dysregulation at internal medicine.
7	17	Female	EM+TTH	Missing school	-	Lomerizine	Galcanezumab	10	10	64	30	30	64	3m, stopped. Treated as orthostatic dysregulation at internal medicine.
8-1	17	Female	EM	Detrimental to study	Dysmenorrhea	Kampo (goreisan), low-dose pill	Fremanezumab 675mg	4	4	55	1	1	46	3m, switched to galcanezumab.
8-2	17	Female	EM	Detrimental to study	Dysmenorrhea	Kampo (goreisan), low-dose pill	Switch to galcanezumab	1	1	46	0	0	36	5m
9	17	Male	EM	Detrimental to playing sports	-	Amitriptyline	Galcanezumab	5	5	62	2	0	40	6m
10	17	Female	EM	Detrimental to study	-	Propranolol	Galcanezumab	4	4	55	0	0	36	4m

**Figure 1:**
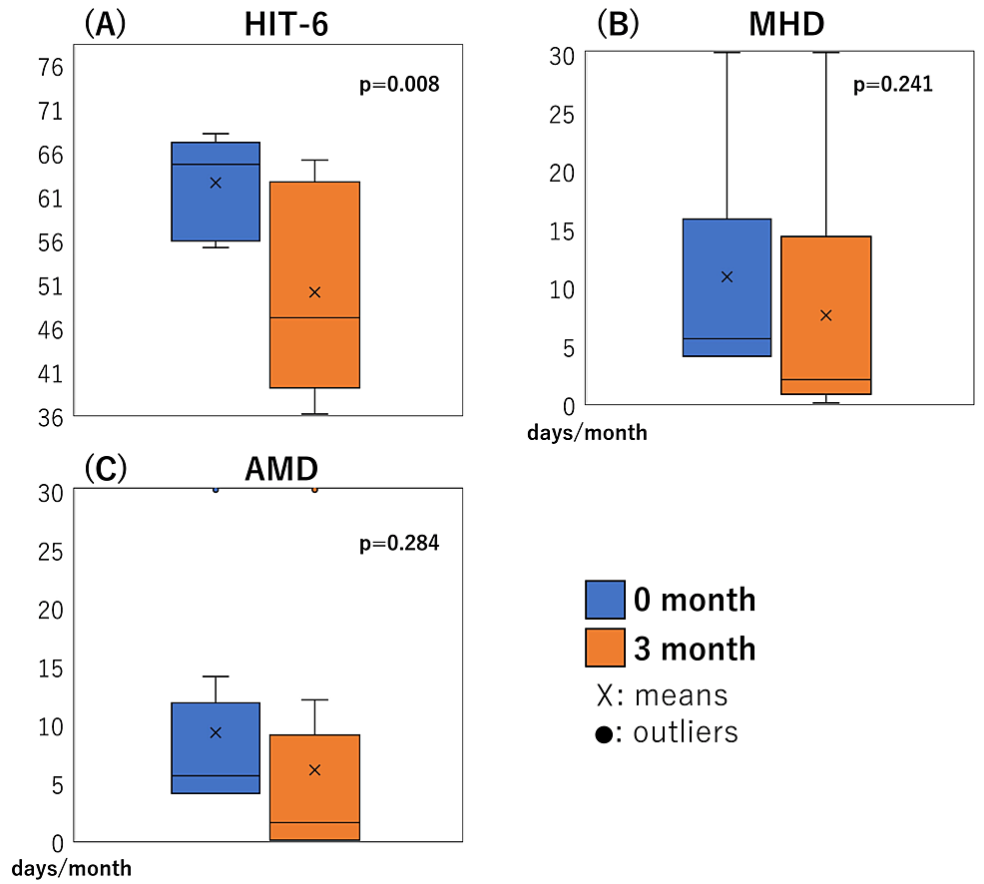
Treatment efficacy boxplots of the Headache Impact Test-6 (HIT-6) scores (A) boxplots of the Headache Impact Test-6 (HIT-6) score, (B) monthly headache days (MHD), and (C) monthly acute medication intake days (AMD) before treatment (0 months, blue) and after three months (orange). HIT-6 score (A) significantly improved after treatment (p=0.008). However, MHD (p=0.241) and AMD  (p=0.284) did not improve (B and C). ☓ - mean, points - outliers

The median HIT-6 before and three months after treatment was 63 (46-68) and 44 (36-65), respectively. MHD before and three months after treatment was 5.5 (1-29) and 1.5 (1-30), respectively, and the median AMD was 5.5 (1-30) and 1 (0-30), respectively. A significant reduction of HIT-6 was observed at three months (p=0.008). However, no significant reductions were confirmed in MHD (p=0.241) and AMD (p=0.284) (Figure 1). Six (60%) of the ten patients experienced therapeutic effectiveness. Patients with missing school as the chief obstacle to life due to headaches seemed ineffective compared to those with other obstacles (p=0.048). There were no side effects of CGRP-mABs.

## Discussion

We herein described the ten adolescent migraine patients treated with CGRP-mABs. HIT-6 score significantly decreased at three months, and six (60%) of the ten patients experienced therapeutic effectiveness measured by HIT-6. Patients with missing school as the chief obstacle to life due to headaches seemed ineffective compared to those with other obstacles. In addition, there were no side effects of CGRP-mABs.

Previous reports on CGRP-mABs for adolescents

The three types of CGRP-mABs, galcanezumab, fremanezumab, and erenumab, used in this case series, were developed for the treatment of migraine in adults and have not been studied for the use of patients aged under 18 years. Few studies on adolescents and CGRP-mABs were reported, but there are two reports on the use of CGRP-mABs for adolescents previously.

Greene et al. [12] reported 112 children and adolescents who received CGRP-mABs to treat their chronic headache disorders. The mean age at the first dose was 15.9 years. They had CM (n=94), new daily persistent headaches (n=12), and persistent post-traumatic headaches (n=6). At baseline, the mean number of headache days per month was 26.9 (n=109). At the first follow-up visit (mean 2.7 months), the headache frequency improved to -2.0 days compared with the baseline. Significant benefit was defined as having at least one of four items; 1) a decrease in headache frequency by at least one-third for at least one month, 2) a decrease in headache intensity by at least one-third for at least one month, 3) a decrease in headache duration by at least one third for at least one month, or 4) notes document a "significant" or "substantial" improvement in headache for at least one month. The significant benefit was confirmed by 29.5% of patients at the first follow-up visit (n=33/112) and 30.1% (n=22/73) at the second follow-up visit (mean 4.6 months). Injection site reactions in 17.0% (n=19) and constipation in 8.0% (n=9) were found as the most common side effects. Five patients (4.5%) discontinued because of side effects.

Zhao et al. [13] reported adolescents treated with erenumab who were aged 15 to 18 years; two had EM, and four had CM. Four of the six adolescent patients treated had a history of prior treatment failure with three or more migraine oral prophylaxis. At baseline, the mean monthly migraine days was 22.3 days. The mean monthly migraine days improved to 17.5 days at the follow-up visit. Favorable response and tolerability to erenumab were confirmed in one EM and one CM patient out of the six adolescent patients, despite the heterogeneous sex, race, and migraine type.

Compared to the previous two reports, our case series consisted of relatively mild or moderate migraine patients. The therapeutic effect was confirmed as 60% among those. Therefore, CGRP-mABs may be more effective for mild or moderate migraine adolescents than for severe ones. Those reports and our case series suggested that CGRP-mABs may be one of the effective prophylaxis for adolescents. Further real-world data is required.

Current recommendations of CGRP-mABs for children and adolescents

In 2018, the American Headache Society published the guideline about the CGRP-mABs use for children and adolescents as an expert opinion [9]. The guideline describes caution of CGRP-mABs and the association between CGRP and mental/physical growth and development. It mentions the facts that CGRP relates to central nervous system maturation, maintenance of pregnancy and fetal development, bone formation, ossification, modulating glucose-stimulated insulin release, immune response, and vasodilating. It also shows the recommendation of CGRP-mABs for children and adolescents (Table 2).

**Table 2:** Recommendations of CGRP-mABs in children and adolescents CGRP-mABs - calcitonin gene-related peptide-related monoclonal antibodies, Ped-MIDAS - Pediatric Migraine Disability Assessment

Indications
≥ 8 headache days per month
PedMIDAS score ≥ 30
Failure of ≥2 preventive therapies (pharmacologic, nutraceutical, and/or non-pharmacologic)
Post-pubertal adolescent, or pre-pubertal child in selected cases
Contraindications
Disturbed blood-brain barrier (e. g. recent history of meningitis, recent neurosurgery)
Severe cardiovascular disease, stroke
Pregnancy, planned pregnancy, or breast-feeding
Monitoring
Pubertal status
Bone health, consider checking vitamin D status
Linear growth
Weight and body mass index
Infections
Pregnancy status

In the guideline, CGRP-mABs should be considered for post-pubertal adolescents suffering from relatively frequent migraine with moderate or severe migraine-related disabilities. It also mentions that CGRP-mABs should be used for two months first, continued until the therapeutic goal is reached, and then terminated as appropriate [9].

The patients in our case series had fewer headache days than in previous reports [12,13]. In such relatively mild migraine cases, CGRP-mABs may be more likely to show a favorable therapeutic effect. Also, CGRP-mABs were less efficacy for the patients whose chief obstacles were missing school. It was suggested that CGRP-mABs were effective in patients with mild migraine and ineffective in those missing school. Therefore, the indication of CGRP-mABs in adolescents may require not only previous guidelines based on headache days and disability scoring but also real-life clinical considerations.

In light of this real-world evidence, rather than simply considering the high-frequent number of days to determine the indication for CGRP-mABs in adolescent patients, CGRP-mABSs administration can be considered even if the number of headache days is low. It may also be worthwhile to consider CGRP-mABs based not on the Pediatric Migraine Disability Assessment (Ped-MIDAS) total score but rather on the subdivision scores in Ped-MIDAS, representing specific obstacles to life. Furthermore, considering that the patient is still in the middle of developmental growth and CGRP-mABs' unknown side effects, termination of CGRP-mABs should be considered once some efficacy is achieved or a certain period of time passes. Discontinuation of CGRP-mABs should be considered in the future, even for our patients who are responding well to CGRP-mABs.

Limitations

The present results were based on a small number of cases at a single institution in Japan. Therefore, it remains unknown whether the results will be similar for adolescent migraine patients overseas. CGRP-mAB injections may have triggered the patients to keep a headache diary and to think about their health more carefully. It is also possible that other prophylactic drugs may have had a delayed effect. Further investigation with a large population is needed. Also, the patients with missing school might have been a coincidence of psychiatric symptoms in addition to migraines. Therefore, CGRP-mABs possibly could not perform therapeutic effects.

Time has passed since the CGRP-mABs were put on the market, and real-world data is accumulating. Further investigation into the use of CGRP-mABs in children and adolescent migraine patients [14] is warranted, although unknown side effects for those by CGRP-mABs should be considered.

## Conclusions

We herein described the ten adolescent migraine patients treated with CGRP-mABs. HIT-6 score significantly decreased at three months, and six (60%) of the ten patients experienced therapeutic effectiveness measured by HIT-6. Patients with missing school as the chief obstacle to life due to headaches seemed ineffective compared to those with other obstacles. There were no side effects of CGRP-mABs. Now several trials have been ongoing to test the efficacy of CGRP-mABs for adolescents. Urgent evidence accumulation is needed about CGRP-mABs for adolescents.
